# HHLA2-KCTD12 axis: a potential mechanism regulating the biological functions of laryngeal squamous cell carcinoma

**DOI:** 10.3389/fonc.2026.1898263

**Published:** 2026-10-08

**Authors:** Yue Yu, Jianqiang You, Xiaoye Wang, Haixiang Xue, Haijun Chen, Wenjing Li

**Affiliations:** Department of Otorhinolaryngology Head and Neck Surgery, the Third Affiliated Hospital of Soochow University (Changzhou First People’s Hospital), Changzhou, Jiangsu, China

**Keywords:** HHLA2, immune checkpoint, KCTD12, laryngeal squamous cell carcinoma, TNF signaling pathway

## Abstract

**Background:**

Accumulating evidence suggests that aberrant HHLA2 expression is closely implicated not only in tumorigenesis, disease progression, and metastatic spread, but also in the intricate modulation of the tumor immune microenvironment. Nevertheless, the biological functions of HHLA2 and the molecular mechanisms underlying its action in laryngeal squamous cell carcinoma (LSCC) remain unexplored.

**Materials and methods:**

HHLA2 expression was systematically assessed in LSCC tumor specimens and matched adjacent tissues using multiplex immunofluorescence (mIHC). The functional consequences of HHLA2 silencing in LSCC cells were evaluated through a series of *in vitro* assays, including CCK-8–based proliferation analysis, wound-healing (scratch) assays, and invasion assays. Transcriptomic sequencing integrated with public database mining was subsequently performed to identify key target genes potentially governed by HHLA2 in regulating LSCC biological behavior. Furthermore, Gene Ontology (GO), Kyoto Encyclopedia of Genes and Genomes (KEGG) and Gene Set Enrichment Analysis (GSEA) pathway analyses were conducted to delineate the signaling networks and molecular pathways through which HHLA2 might exert its regulatory effects in LSCC.

**Results:**

HHLA2 expression was significantly elevated in LSCC tumor tissues, and associated with poor prognosis in patients. Functional experiments revealed that suppression of HHLA2 leads to marked inhibition of LSCC cell proliferation, migration, and invasion, underscoring its essential role in sustaining malignant phenotypes. Mechanistically, we identified potassium channel tetrameric domain 12 (KCTD12) as a pivotal target of HHLA2 in mediating these oncogenic processes. Importantly, forced overexpression of KCTD12 effectively counterbalanced the inhibitory effects induced by HHLA2 depletion, thereby restoring proliferative, migratory, and invasive potential to LSCC cells. Furthermore, pathway enrichment analyses indicated that the TNF—NF-κB signalling pathway might serve as a key conduit through which HHLA2 orchestrated both tumor cell functions and immune regulatory mechanisms. The HHLA2-KCTD12 axis may regulate the phenotype of LSCC cells and reshape the tumor immune microenvironment through the TNF—NF-κB signaling pathway.

**Conclusions:**

HHLA2 enhanced the proliferative, migratory, and invasive capacities of LSCC cells and might contribute to immune microenvironment remodeling via modulation of TNF—NF-κB signaling through the HHLA2–KCTD12 axis.

## 1 Introduction

Laryngeal cancer is the 11th most common malignant tumor worldwide, accounting for about 5% of all malignancies (1, 2). In recent years, its incidence has continued to rise, representing a growing threat to patient health and survival (3). Although advances in treatment strategies have modestly improved overall survival (OS), the recurrence and metastasis rates remain as high as 30%–40% (4). At present, surgery remains the primary therapeutic option, often combined with radiotherapy or chemotherapy. However, owing to the unique physiological functions of the larynx, surgical interventions, whether partial or total laryngectomy, can severely impair speech, respiration, and swallowing, thereby diminishing both quality of life and long-term outcomes (5). Given the high recurrence rates, delayed diagnosis, functional impairments following surgery, and the adverse effects associated with chemotherapy, elucidating the molecular pathogenesis of laryngeal cancer and identifying effective therapeutic targets have become pressing clinical priorities.

The B7 family plays a pivotal role in maintaining the delicate balance between effective immune activation and the prevention of autoimmunity. This family comprises 10 members, including the well-characterized B7-H1 (PD-L1), B7-1 (CD80), B7-2 (CD86), B7-DC (PD-L2), B7-H2 (CD275), B7-H3 (CD276), B7-H4, B7-H5, B7-H6, and B7-H7 (human endogenous retrovirus-H long terminal repeat-associating 2, HHLA2) (6). These molecules are membrane-anchored protein ligands that interact with CD28 family receptors on immune cells, thereby regulating immune responses through either costimulatory or inhibitory pathways (7). Consequently, therapeutic blockade of B7 family members has been shown to enhance antitumor immunity. Among them, HHLA2, the most recently identified member, has attracted particular interest owing to its dual roles in tumor biology and the immune microenvironment. While PD-1/PD-L1 has been extensively studied, evidence indicates that in certain malignancies such as renal clear cell carcinoma (KIRC) and intrahepatic cholangiocarcinoma, HHLA2 expression exceeds that of PD-L1, and its expression is independent of PD-L1, suggesting that the mechanisms by which the immune system regulates HHLA2 and PD-L1 are entirely distinct (8–11). These findings imply that therapeutic inhibition of HHLA2 and its signalling pathways may provide a novel strategy for cancer immunotherapy.

HHLA2 (also referred to as B7H7 or B7y) is a type I transmembrane glycoprotein and the most recent addition to the B7 ligand family (12). Structurally, HHLA2 resembles CD80/CD86, with its gene located primarily on chromosome 3 (13). A distinguishing feature of HHLA2 is the presence of three extracellular immunoglobulin domains (IgV, IgC, and IgV) (11). By contrast, B7-H3 contains two IgV and two IgC domains, whereas most other B7 family members typically harbour only one IgV and one IgC domain (14). HHLA2 is broadly expressed in a wide range of human tumors; however, its biological effects differ across cancer types, possibly due to the dynamic changes in the tumor microenvironment (TME) during disease progression, which may contribute to these differences among different cancer types (15).

This study sought to elucidate the specific mechanisms through which HHLA2 regulates the biological behavior of human laryngeal squamous cell carcinoma (LSCC) and to clarify the critical role of its aberrant expression in driving LSCC progression.

## 2 Materials and methods

### 2.1 Patient data and tissue specimens from LSCC

LSCC tissue specimens were obtained from patients who underwent treatment at the Third Affiliated Hospital of Soochow University between 01/01/2010 and 30/12/2022. All enrolled patients had undergone either laryngeal tumor resection or biopsy, with histopathological diagnoses independently confirmed by two experienced pathologists, Formalin-fixed paraffin-embedded tissue samples were collected, and representative regions of interest were selected to construct tissue microarrays. Ultimately, 79 patients were enrolled in this study. A total of 110 cases of LSCC data were downloaded from the TCGA database. The scRNA-seq data of LSCC were extracted from the GSE234933 dataset and polygenic prediction data were obtained from the GSE27020 dataset in the GEO database.

### 2.2 Multiplex immunohistochemistry

Tissue sections were dewaxed in xylene, rehydrated through a graded ethanol series, and fixed in 10% neutral buffered formalin (NBF) for 10 min. Staining was then performed. At the start of each staining cycle, slides were subjected to heat-induced antigen retrieval in Tris-EDTA buffer. After blocking nonspecific proteins for 10 min, primary antibodies were sequentially incubated at 37 °C for 60 min. This was followed by incubation with horseradish peroxidase (HRP)-conjugated secondary antibodies and tyramide signal amplification (TSA) using the Opal System. Microwave treatment was applied at the end of each staining cycle to remove the antibody-TSA complex. Finally, nuclei were counterstained with 4′,6-diamidino-2-phenylindole (DAPI, Sigma-Aldrich) for 8 min, and the slides were mounted with coverslips. Whole-slide imaging was performed using a Pannoramic MIDI scanner (3DHISTECH).

### 2.3 Quantitative PCR

Total RNA was extracted from TU686 and TU177 cells (5 × 10^6^ cells/mL) using TRIzol^®^ reagent (Thermo Fisher Scientific, Inc.) according to the manufacturer’s protocol. The extracted RNA was then reverse-transcribed into cDNA using the PrimeScript RT Reagent Kit (Takara Bio, Inc.). qPCR analysis of gene expression was performed on an ABI PRISM 7000 Sequence Detection System (Applied Biosystems; Thermo Fisher Scientific, Inc.) following the manufacturer’s instructions. The thermocycling conditions were as follows: initial denaturation at 95 °C for 7 min, followed by 40 cycles of 95 °C for 15 sec and 60 °C for 30 sec, and a final extension at 72 °C for 30 sec. Relative gene expression levels were determined using the 2^−ΔΔCq^ method (16), with GAPDH as the internal control. Each experiment was performed in triplicate. The primer sequences used in this study are listed in (**Table S1**).

### 2.4 Cell lines and culture conditions

Human LSCC cell lines TU177 and TU686 were obtained from the Chinese Academy of Sciences Cell Bank, and 293T cells were purchased from Takara Bio. All cell lines were maintained in a humidified incubator at 37 °C with 5% CO_2_, using the appropriate culture medium specified by the supplier.

### 2.5 Western blotting analysis

A lysis cocktail containing protein inhibitor, phosphatase inhibitor, and RIPA buffer (Seven) was prepared at a ratio of 1:1:100. Cells were lysed on ice with this mixture for 2 h, followed by centrifugation at 12,000 × g at 4 °C for 30 min. The supernatant was collected, and protein concentration was determined using a BCA assay kit (Beyotime Biotechnology). For sample preparation, 5× loading buffer (Seven) was added to the protein solution at a 1:4 ratio, and the mixture was heated at 95 °C for 10 min. Proteins were then separated on 10% SDS-PAGE gels and transferred onto PVDF membranes (Millipore). Membranes were blocked with 5% skimmed milk in Tris-buffered saline containing 0.1% Tween-20 for 2 h at room temperature. After blocking, membranes were incubated with primary antibodies against GAPDH at 4 °C for 12 h. Following washes, membranes were incubated with the appropriate secondary antibodies at room temperature for 1.5 h. Immunoreactive protein bands were visualized using enhanced chemiluminescence (Thermo Fisher Scientific).

### 2.6 CCK-8 assay

Cell suspensions from each group, at a density of 2 × 10^4^ cells/mL, were seeded into 96-well plates (100 μL per well). Plates were incubated at 37 °C with 5% CO_2_, and observations were made at 0, 12, 24, 48, 72, and 96 h. At each time point, 10 μL of CCK-8 reagent was added under light-protected conditions, followed by incubation at 37 °C for an additional 1 h. Absorbance at 450 nm was measured using a microplate reader, and cell viability was calculated accordingly.

### 2.7 Wound-healing assay

For the wound-healing assay, 5 × 10^5^ cells/mL from each experimental group were seeded into 6-well plates and incubated at 37 °C with 5% CO_2_ for 16~24 h to achieve a confluent monolayer. The culture medium was then replaced with serum-free medium and incubated for 4 h to minimize cell proliferation, thereby isolating migration effects in the subsequent scratch assay. Linear scratches were introduced using a 10-μL pipette tip, generating 3~5 parallel lines per well. Following three gentle washes with PBS to remove detached cells, fresh serum-free medium was added, and cells were allowed to migrate for 24 h under standard culture conditions. Migration distances were captured via microscopy and quantitatively analyzed using ImageJ software.

### 2.8 Transwell invasion assay

Cell invasive capacity was evaluated using Transwell chambers (Millipore, Billerica, USA). Transfected cells (2.5 × 10^4^) were seeded in the upper chamber containing serum-free medium, while the lower chamber was filled with DMEM supplemented with 10% FBS. The upper surface of the membrane was pre-coated with Matrigel (BD Biosciences, Franklin Lakes, NJ, USA) to mimic the extracellular matrix. After 24 h, cells that had traversed the membrane were fixed, stained with crystal violet, and enumerated under an inverted light microscope at ×100 magnification, counting 10 randomly selected fields per sample to ensure statistical robustness.

### 2.9 Functional enrichment analysis

GO, GSEA and KEGG analyses were conducted using the “clusterProffler” “fgsea” “org.Hs.eg.db” and “enrichplot” packages in R to compare differential signaling pathways and biological processes.

### 2.10 Statistical analysis

All statistical analyses were performed using SPSS 26.0, with graphical representations generated in GraphPad Prism 10.0. Comparisons between two groups employed Student’s t-test, while multiple group comparisons were conducted via one-way ANOVA followed by Tukey’s *post hoc* test. *P*-value < 0.05 was considered statistically significant.

Differential expression analysis was conducted using the edgeR package. *P*-values were calculated for each gene and adjusted for multiple comparisons to control the false discovery rate (FDR), yielding Q-values. Fold changes (FC) were derived from FPKM (fragments per kilobase of transcript per million mapped fragments) values and expressed as log_2_(FC), where FC ≥ 2 indicates upregulation and FC ≤ 0.5 indicates downregulation. Genes were considered significantly differentially expressed if they met the criteria of Q < 0.05, FC ≥ 2, and FPKM > 1 in at least one experimental group. Survival analysis using the “survival” and “survminer” packages in R to calculate the optimal cutoff value and plot Kaplan-Meier survival curves.

## 3 Results

### 3.1 HHLA2 is highly expressed in LSCC and positively correlates with Ki67 and CD34

UMAP-based dimensionality reduction analysis of throat scRNA-seq data (GSE234933) enabled the identification of five major cell populations, namely tumor cells, endothelial cells, fibroblasts, myeloid cells, and lymphocytes. Subsequent detailed examination of the immune-related gene HHLA2 revealed that its expression was predominantly localized within tumor cell populations, with minimal or essentially undetectable expression in the remaining cell types. This observation was further reinforced by DotPlot analysis, which demonstrated that HHLA2 exhibited both the highest expression levels and the greatest proportion of positive cells in the tumor cell population, whereas fibroblasts, endothelial cells, myeloid cells, and lymphocytes displayed substantially lower or absent expression (**Figure 1A**). Collectively, these findings indicated that HHLA2 expression exhibited marked cell type specificity, being primarily associated with tumor cells.

**Figure 1:**
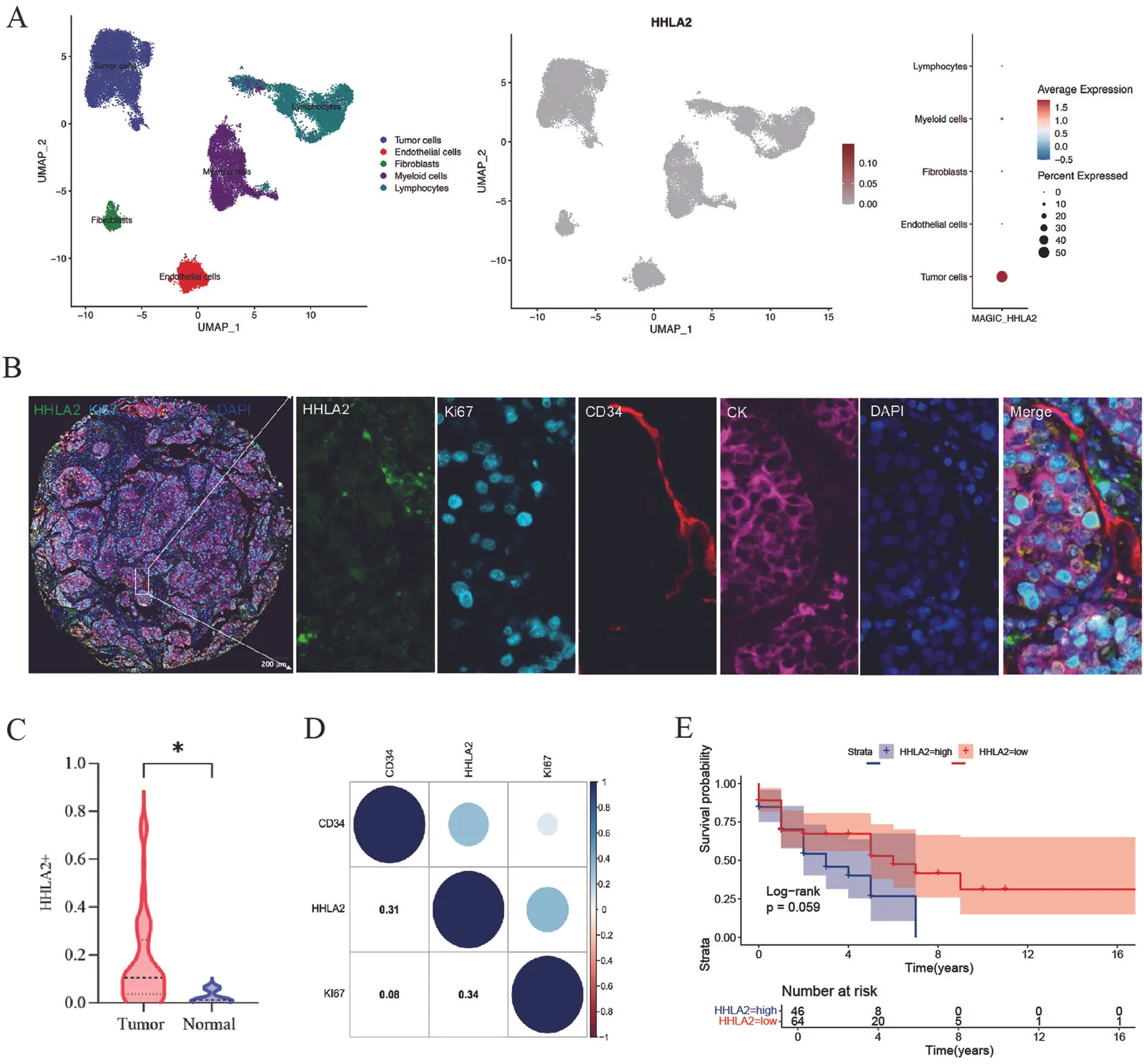
Single-cell and tissue-level analysis of HHLA2 expression in LSCC. **(A)** UMAP plot illustrating the clustering of single-cell RNA sequencing (scRNA-seq) data from LSCC. UMAP plot depicting the expression distribution of HHLA2 in the scRNA-seq dataset of LSCC. Scatter plot showing the average expression level and percentage of cells expressing HHLA2 in the scRNA-seq data. (Data were obtained from the GSE234933 dataset) **(B)** Multiplex immunofluorescence staining demonstrating specific expression of HHLA2, Ki67, and CD34 in tumor tissues. **(C)** Comparison of HHLA2 expression between tumor and adjacent non-tumor tissues, analyzed by the Mann–Whitney U test, indicating higher expression in tumor tissues (Data were obtained from 79 patients). **(D)** Spearman correlation analysis revealed a significant positive association between HHLA2 expression and the expression levels of Ki67 and CD34 among the 79 patients. **(E)** Kaplan-Meier survival analysis of HHLA2 expression and prognosis in 110 patients with LSCC from the TCGA database.

To validate these observations at the tissue level, mIHC was performed on LSCC tissues as well as on adjacent non-tumor tissues. The results showed that HHLA2 expression was largely localized to the cell membrane of tumor cells (**Figure 1B**), and quantitative analysis revealed a significantly higher expression of HHLA2 in tumor tissues compared with adjacent non-tumor tissues, with the difference reaching statistical significance (*P* < 0.05) (**Figure 1C**). These findings provided further evidence that HHLA2 expression was predominantly concentrated in tumor tissue.

Given that Ki67 serves as a marker of tumor cell proliferation and CD34 is associated with tumor-related angiogenesis, we next examined the expression patterns of these two markers in LSCC tumor tissues using mIHC staining. The results demonstrated that both Ki67 and CD34 exhibited distinct and specific signal localization within the tumor tissue samples (**Figure 1B**). Furthermore, Spearman correlation analysis revealed that HHLA2 expression was significantly and positively correlated with the expression of Ki67 and CD34 (**Figure 1D**), suggesting a potential mechanistic link between HHLA2 expression and both proliferative activity and angiogenic processes within LSCC. Extract FPKM data and clinical data of 110 patients with laryngeal squamous cell carcinoma from the TCGA database. Using the R packages “survival” and “survminer”, calculate the “cutoff” value and perform Kaplan-Meier survival analysis to evaluate the relationship between HHLA2 expression and the prognosis of patients with laryngeal squamous cell carcinoma. It was found that higher HHLA2 expression was associated with worse overall survival (OS) in these patients (**Figure 1E**).

### 3.2 Downregulation of HHLA2 impairs proliferation, migration, and invasion of LSCC

Two LSCC cell lines, TU177 and TU686, were selected to investigate the functional role of HHLA2. Stable HHLA2-depleted cell lines were generated via lentiviral packaging and transfection, and the efficiency of gene silencing was validated using qPCR and Western blotting analyses, confirming the successful establishment of cell lines with sustained HHLA2 downregulation (**Figures 2A–D**).

**Figure 2:**
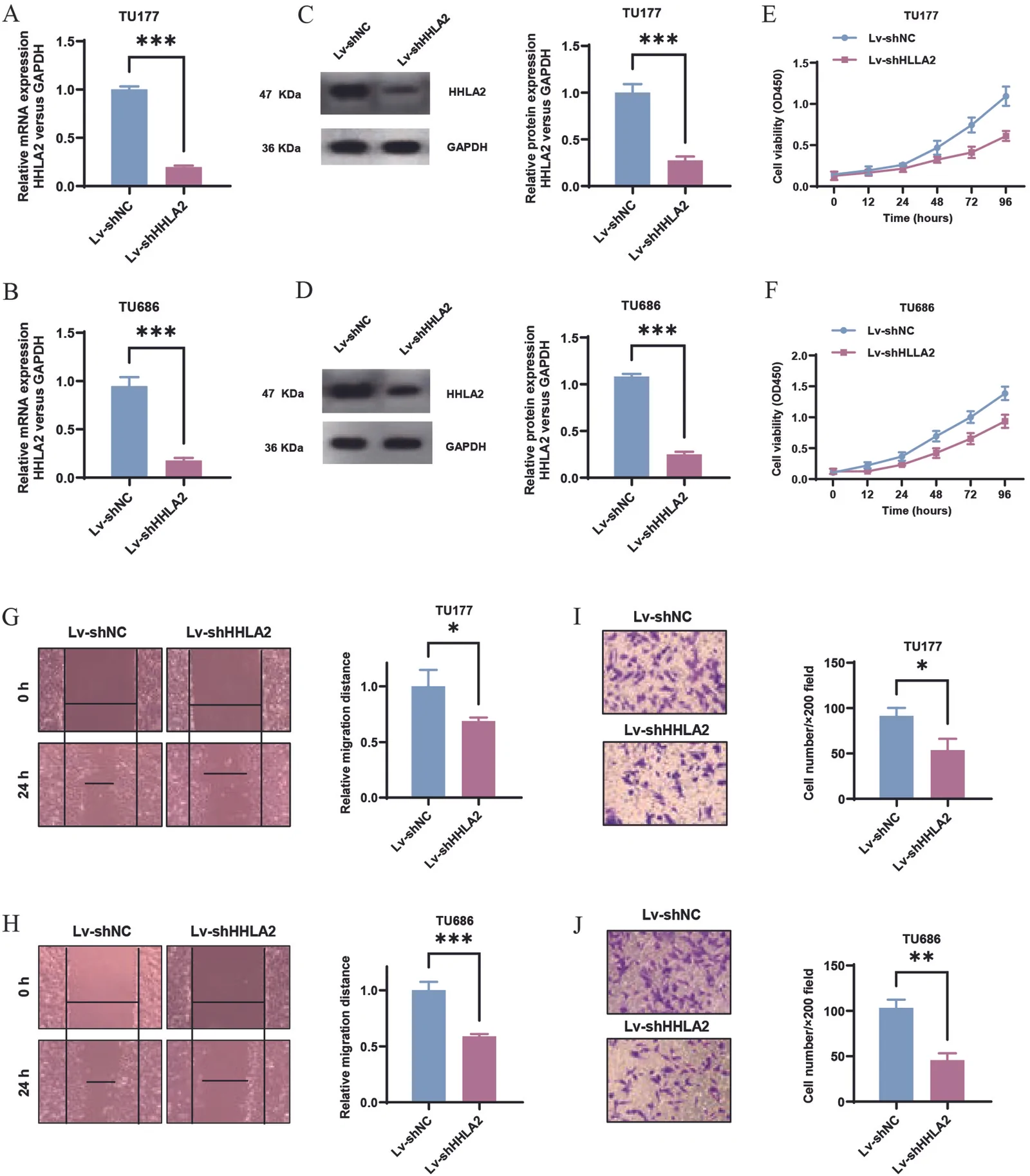
Real-time PCR and Western blotting validation of HHLA2 downregulation efficiency. **(A, B)** qPCR analysis confirming HHLA2 knockdown, showing that HHLA2 mRNA levels in the Lv-shHHLA2 experimental group were significantly lower than those in the Lv-shNC control group. **(C, D)** Western blotting analysis demonstrating that HHLA2 protein expression was markedly reduced in the Lv-shHHLA2 experimental group compared to the Lv-shNC control group. **(E, F)** CCK-8 proliferation assay results indicating that the proliferative capacity of Lv-shHHLA2 cells was significantly reduced relative to the Lv-shNC control group. **(G, H)** Wound-healing assay showing that the migratory ability of Lv-shHHLA2 cells was significantly decreased compared with the Lv-shNC control group. **(I, J)** Transwell invasion assay results demonstrating that the invasive ability of Lv-shHHLA2 cells was significantly lower than that of Lv-shNC control cells following HHLA2 knockdown.*P < 0.05, **P < 0.01, ***P < 0.001.

Functional evaluation of these cell lines revealed that HHLA2 suppression markedly impaired tumor cell behavior. CCK-8 proliferation assays demonstrated that HHLA2 knockdown significantly reduced the proliferative capacity of both TU177 and TU686 cells (**Figures 2E, F**). Consistently, wound healing assays indicated a substantial inhibition of migratory ability in HHLA2-deficient cells relative to control cells (**Figures 2G, H**). Moreover, Transwell invasion assays showed that HHLA2 knockdown led to a pronounced decrease in the invasive potential of both cell lines compared with their corresponding controls (**Figures 2I, J**). Collectively, these results indicated that HHLA2 played a critical role in promoting the proliferation, migration, and invasion of LSCC cells, highlighting its potential as a therapeutic target.

### 3.3 Identification of key genes regulated by HHLA2 in modulating LSCC cell function

Volcano plot analysis revealed substantial differences in both upregulated and downregulated genes when comparing TU177-Lv-shHHLA2 with Lv-shNC, as well as TU686-Lv-shHHLA2 with Lv-shNC, highlighting significant transcriptional changes upon HHLA2 knockdown (**Figure 3A**). Subsequent VENNY analysis indicated that the two cell lines, TU177-Lv-shHHLA2 and TU686-Lv-shHHLA2, shared 5,115 genes exhibiting consistent expression trends, suggesting overlapping regulatory effects of HHLA2 suppression (**Figure 3B**).

**Figure 3:**
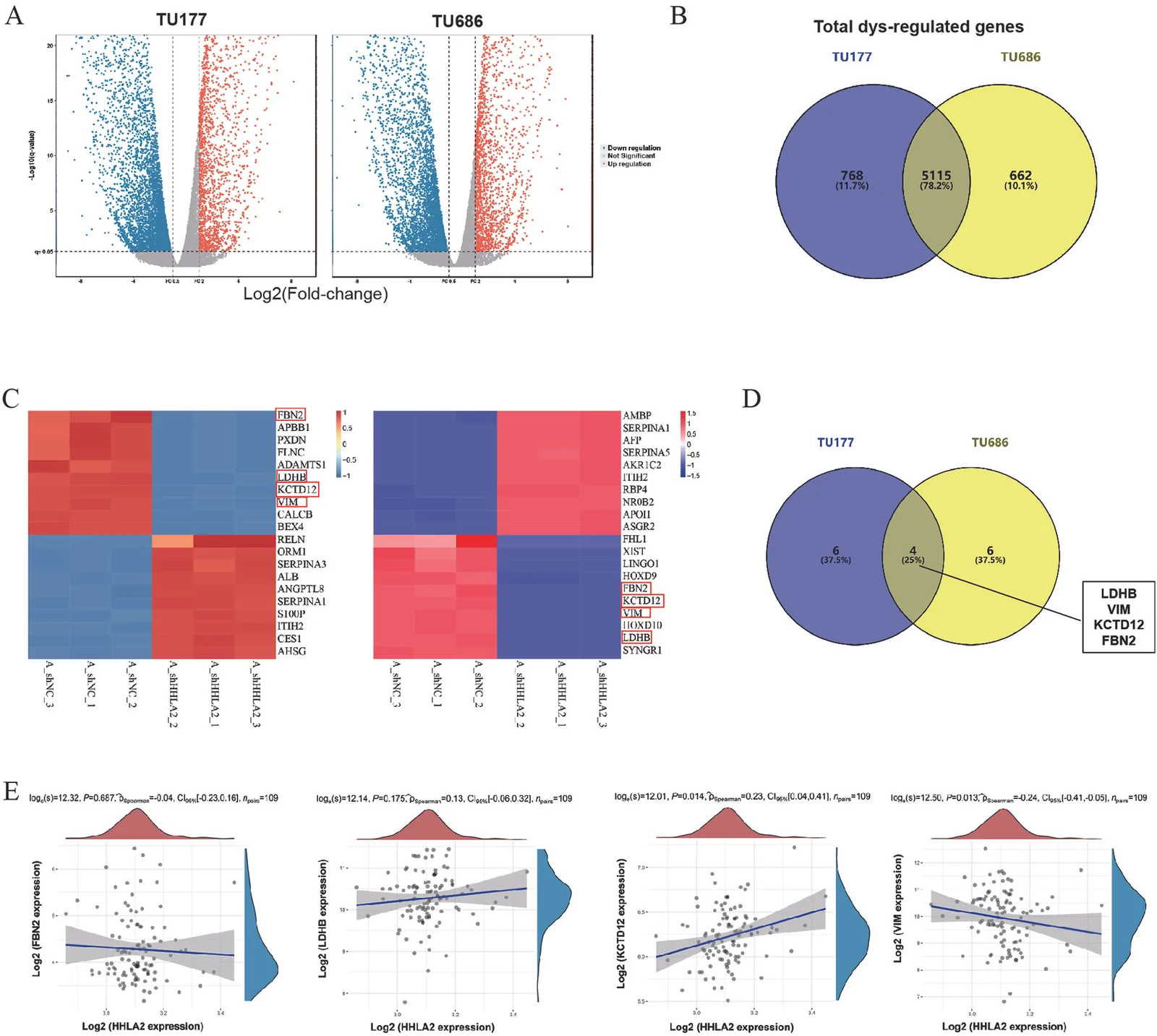
Analysis of key genes modulated by HHLA2 knockdown. **(A)** Volcano plot illustrating the overall distribution of DEGs, with red indicating significantly upregulated genes and blue indicating significantly downregulated genes. **(B)** Venn diagram (VENNY analysis) showing the overlap among multiple gene lists. **(C)** Heatmap of DEGs displaying the overall expression patterns of the top 10 upregulated and downregulated genes. Red represents protein-coding genes with relatively high expression, whereas blue represents those with relatively low expression. **(D)** Venn diagram (VENNY analysis) further validates the top 10 co-downregulated genes in TU177-Lv-shHHLA2 and TU686-Lv-shHHLA2 cell lines. **(E)** Spearman correlation analysis revealing the relationships between HHLA2 and the genes FBN2, LDHB, KCTD12, and VIM. The values presented correspond to the correlation coefficient, P-value, and the method used for calculation (GSE27020).

Cluster analysis of the top 10 upregulated and downregulated genes in TU177-Lv-shHHLA2 and TU686-Lv-shHHLA2 further identified four common downregulated genes, LDHB, VIM, potassium channel tetrameric domain 12 (KCTD12), and FBN2, present among the top 10 downregulated genes in both cell lines (**Figure 3C**). VENNY analysis corroborated these findings, confirming the consistent downregulation of these genes across both cell lines (**Figure 3D**).

Analysis of the GEO dataset GSE27020, encompassing 109 human laryngeal carcinoma tissue samples, was conducted to investigate correlations between HHLA2 and the expression of LDHB, KCTD12, VIM, and FBN2. The analysis revealed a significant positive correlation exclusively between HHLA2 and KCTD12 in laryngeal carcinoma tissues (**Figure 3E**), leading to the selection of KCTD12 as the key target of HHLA2 for further functional studies.

### 3.4 HHLA2 knockdown reduces KCTD12 expression, and stable cell lines with HHLA2 knockdown and KCTD12 overexpression are successfully established

To investigate the effect of HHLA2 on KCTD12 expression, qPCR and Western blotting experiments were performed. LSCC cell lines with HHLA2 knockdown and KCTD12 overexpression were constructed, and the efficiency of both knockdown and overexpression was rigorously validated.

qPCR analysis demonstrated that KCTD12 mRNA levels were significantly decreased in TU177 and TU686 cells following HHLA2 knockdown (**Figures 4A, B**; *P* < 0.01 or *P* < 0.001). Consistently, Western blotting analysis revealed that KCTD12 protein levels were also markedly reduced in these cell lines upon HHLA2 suppression (**Figures 4C, D**; *P* < 0.001 or *P* < 0.01).

**Figure 4:**
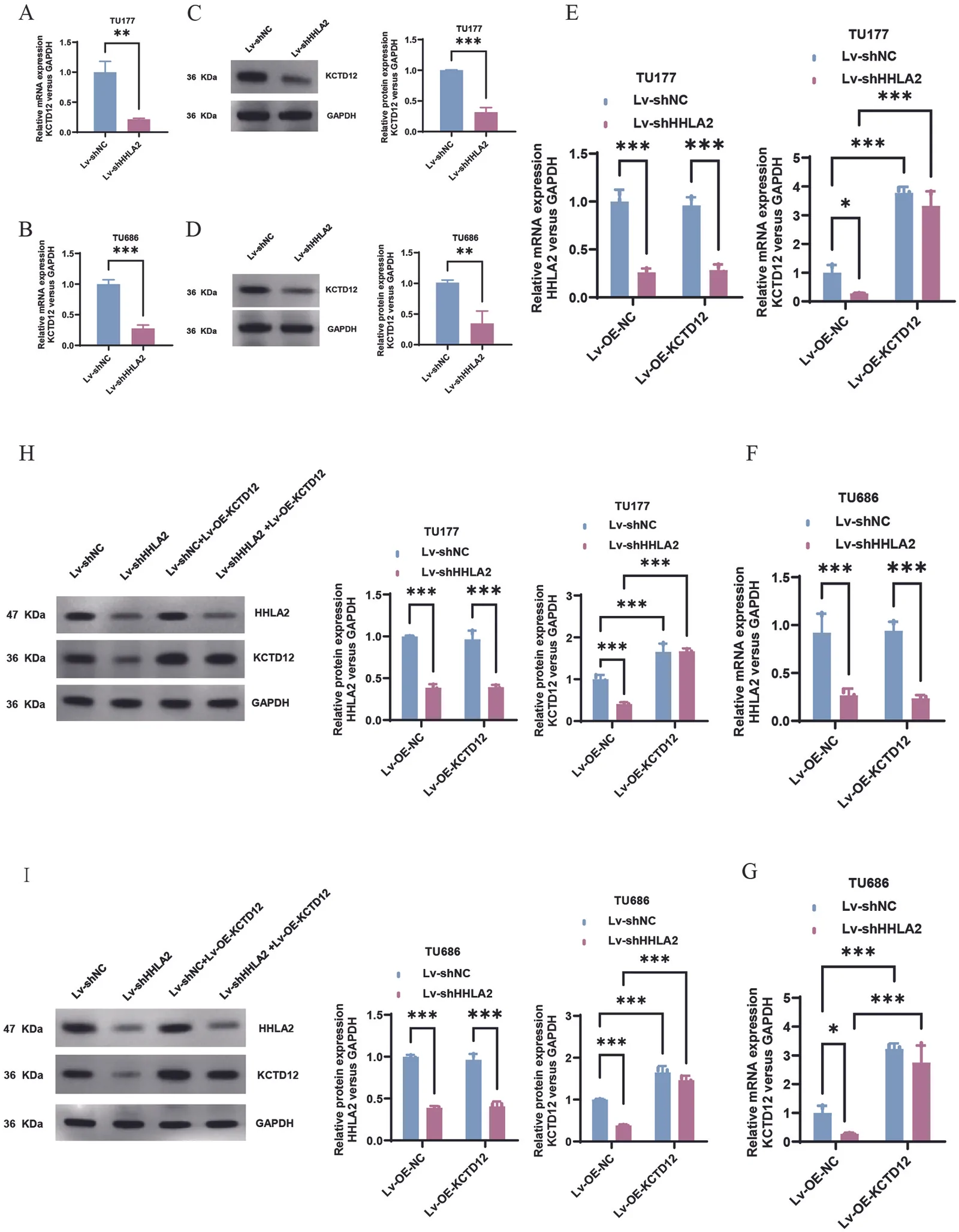
Validation of KCTD12 expression changes following HHLA2 knockdown and efficiency of HHLA2 knockdown and KCTD12 overexpression. **(A, B)** qPCR analysis showing that KCTD12 mRNA levels in the Lv-shHHLA2 group were significantly lower than those in the Lv-shNC control group. **(C, D)** Western blotting analysis demonstrating that KCTD12 protein levels in the Lv-shHHLA2 group were significantly reduced compared to the Lv-shNC control group. *P < 0.05, **P < 0.01, ***P < 0.001. **(E–G)** qPCR validation of HHLA2 knockdown and KCTD12 overexpression, indicating that the Lv-shHHLA2 group exhibited significantly lower HHLA2 mRNA levels compared with the empty vector control group. **(H, I)** Western blotting validation of HHLA2 knockdown, showing that HHLA2 protein expression in the Lv-shHHLA2 group was markedly reduced relative to the empty vector control group. *P < 0.05, **P < 0.01, ***P < 0.001.

Two LSCC cell lines, TU177 and TU686, were employed to generate stable lines with HHLA2 knockdown combined with KCTD12 overexpression through lentiviral packaging and transduction. The efficiency of gene modulation was confirmed using both qPCR and Western blotting analyses, demonstrating the successful establishment of cell lines with stable HHLA2 knockdown and KCTD12 overexpression (**Figures 4E–I**).

### 3.5 Effects of HHLA2 downregulation and KCTD12 overexpression on the proliferative, migratory, and invasive abilities of TU177 and TU686 cell lines

CCK-8 assay results demonstrated that the proliferative capacity of cells in the Lv-shHHLA2 group was markedly reduced compared with the Lv-shNC group. Notably, cells in the Lv-shHHLA2+Lv-OE-KCTD12 group exhibited a significant enhancement in proliferation relative to the Lv-shHHLA2 group, indicating that KCTD12 overexpression effectively reversed the inhibitory effect of HHLA2 downregulation on the proliferative activity of TU177 and TU686 cell lines (**Figures 5A, B**).

**Figure 5:**
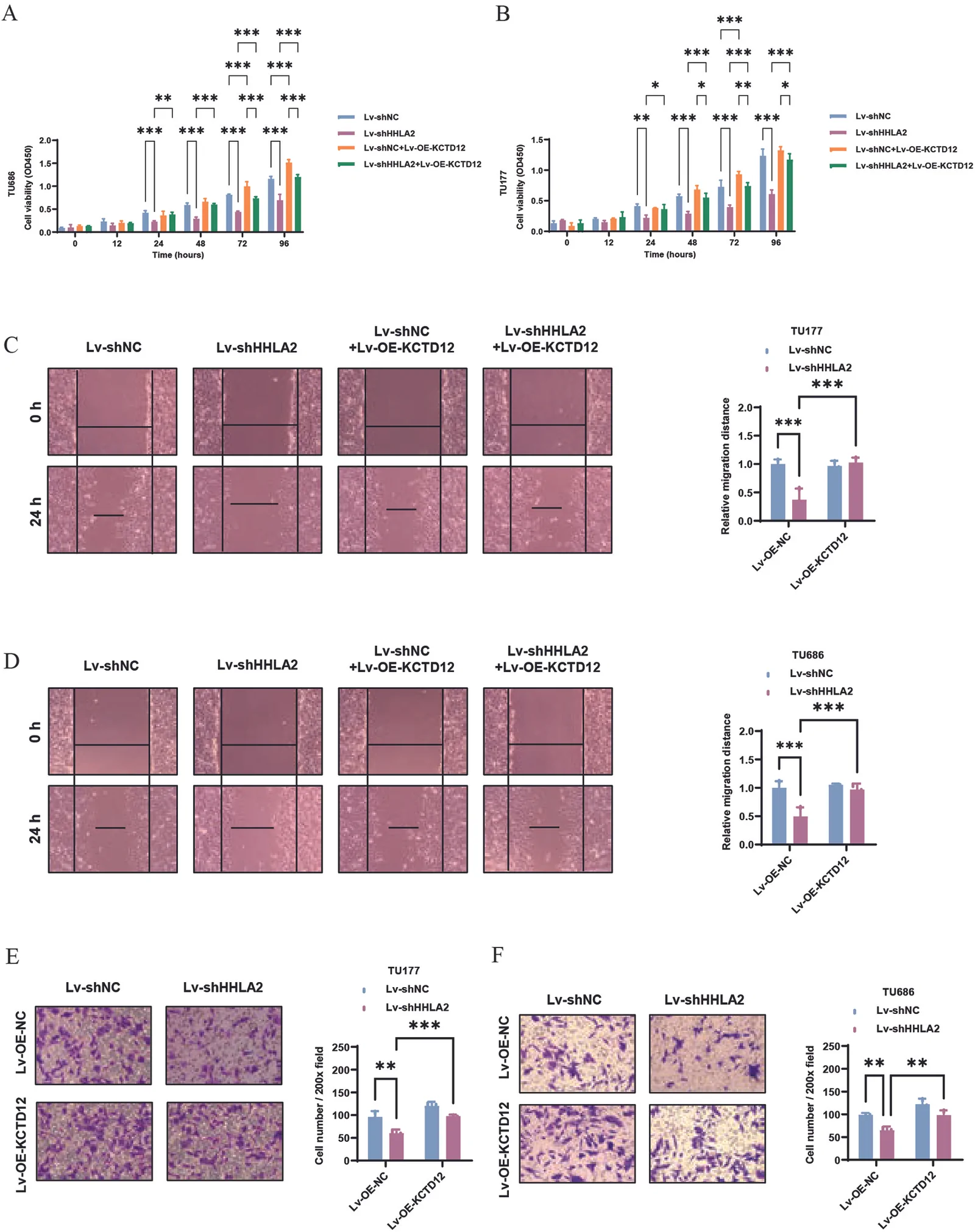
Effects of HHLA2 knockdown and KCTD12 overexpression on the proliferation, migration, and invasion of LSCC cells. **(A, B)** CCK-8 assay results showing that HHLA2 knockdown significantly suppressed the proliferation of TU177 and TU686 cells, whereas KCTD12 overexpression markedly reversed the inhibitory effect of HHLA2 knockdown on cell proliferation. **(C, D)** Scratch assay results demonstrating that HHLA2 knockdown significantly reduced the migratory ability of TU177 and TU686 cells, while KCTD12 overexpression effectively reversed the migration inhibition induced by HHLA2 knockdown. *P < 0.05, **P < 0.01, ***P < 0.001. **(E, F)** Transwell invasion assay results indicating that HHLA2 knockdown significantly impaired the invasive capacity of TU177 and TU686 cells, whereas KCTD12 overexpression substantially restored the invasive ability suppressed by HHLA2 knockdown. *P < 0.05, **P < 0.01, ***P < 0.001.

Similarly, scratch assay results showed that the migratory capacity of cells in the Lv-shHHLA2 group was substantially diminished compared with the Lv-shNC group. In contrast, cells in the Lv-shHHLA2+Lv-OE-KCTD12 group displayed a pronounced increase in migration relative to the Lv-shHHLA2 group, suggesting that KCTD12 overexpression significantly counteracted the suppression of migration induced by HHLA2 knockdown in both TU177 and TU686 cells (**Figures 5C, D**).

Furthermore, Transwell invasion assay results indicated that the invasive potential of cells in the Lv-shHHLA2 group was significantly lower than that of the Lv-shNC group. Importantly, the Lv-shHHLA2+Lv-OE-KCTD12 group showed a remarkable restoration of invasive ability compared with the Lv-shHHLA2 group, demonstrating that KCTD12 overexpression could effectively reverse the inhibitory impact of HHLA2 downregulation on the invasive capacity of TU177 and TU686 cell lines (**Figures 5E, F**).

### 3.6 Gene ontology and gene set enrichment analysis enrichment analyses illustrating the functional pathways altered by HHLA2 downregulation in LSCC

To further elucidate the biological functions of genes within LSCC cells, we conducted GO functional enrichment analysis on DEGs. GO enrichment analysis revealed that HHLA2 was primarily involved in amino acid transport and metabolism, the Wnt signalling pathway, and the epithelial-mesenchymal transition (EMT) signalling pathway (**Figure 6A**).

**Figure 6:**
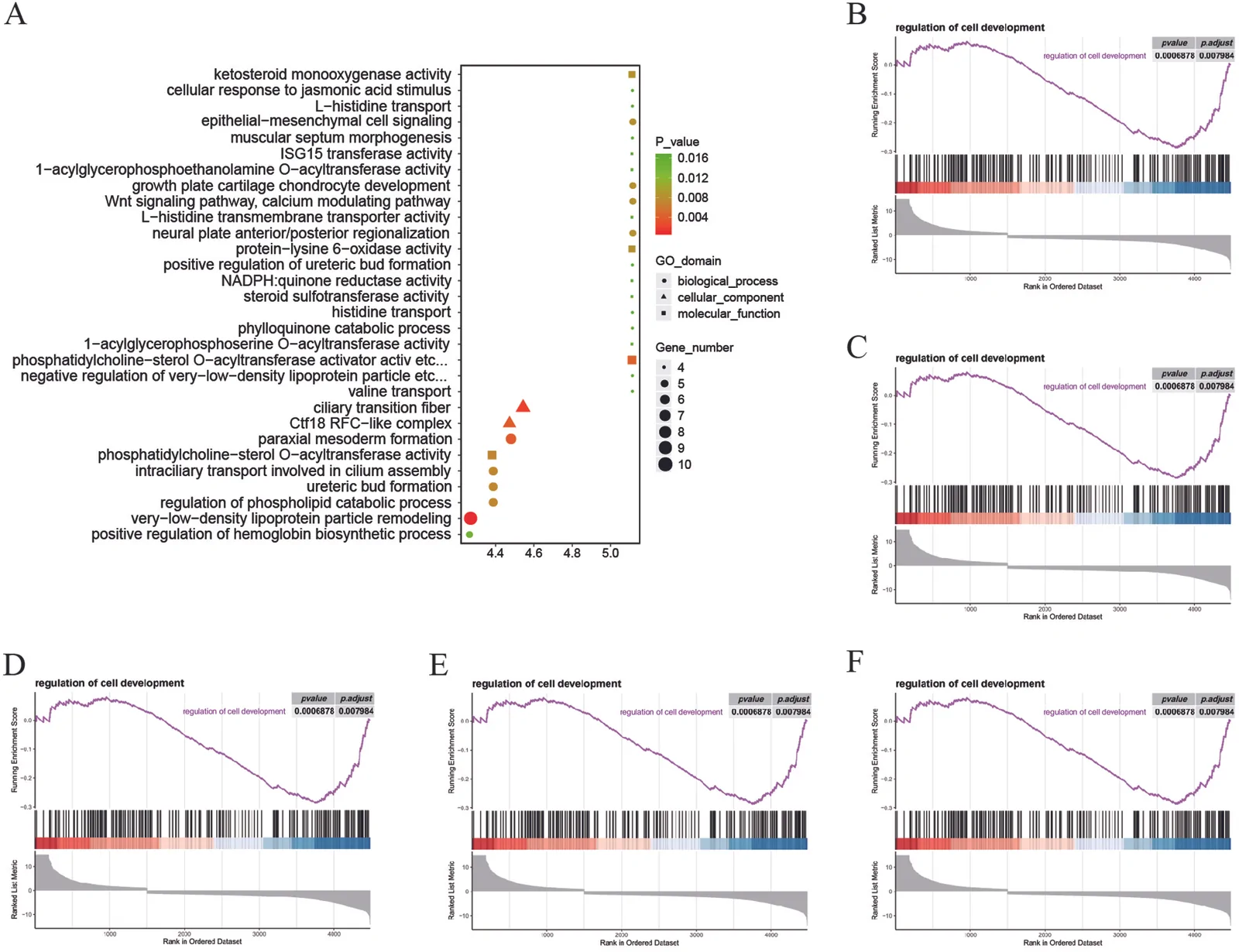
GO and GSEA enrichment analyses of differentially expressed genes (DEGs) following HHLA2 knockdown. **(A)** GO enrichment analysis indicating the functional pathways significantly affected by HHLA2 knockdown. **(B–F)** GSEA enrichment analysis illustrating pathways that were downregulated following HHLA2 knockdown.

GSEA revealed that HHLA2 downregulation led to marked suppression of multiple pathways essential for cellular function. Notably, gene sets associated with cell development, differentiation, migration, angiogenesis, and intercellular Wnt signaling were significantly attenuated (**Figures 6B–F**). Collectively, these results implicated HHLA2 as a critical regulator of tumor cell growth, phenotypic plasticity, and metastatic potential, and further suggested that HHLA2 might contribute to TME remodeling through modulation of angiogenic processes and Wnt pathway activity.

### 3.7 Kyoto encyclopedia of genes and genomes and GSEA enrichment analyses of HHLA2 downregulation in LSCC

KEGG pathway enrichment analysis pinpointed HHLA2 as predominantly involved in the p53, TNF, and Notch signaling pathways, with the TNF pathway showing the highest degree of enrichment (**Figure 7A**). HHLA2 knockdown led to upregulation of pathways associated with immune response, protein processing, cytokine production, inflammatory responses, and T cell activation (**Figures 7B–F**), indicating that reduced HHLA2 expression might enhance tumor cell recognition and immune-mediated activation, thereby promoting inflammatory and immune-related processes. These results collectively suggested that HHLA2 might contribute to remodeling the tumor immune microenvironment, primarily through modulation of TNF signaling.

**Figure 7:**
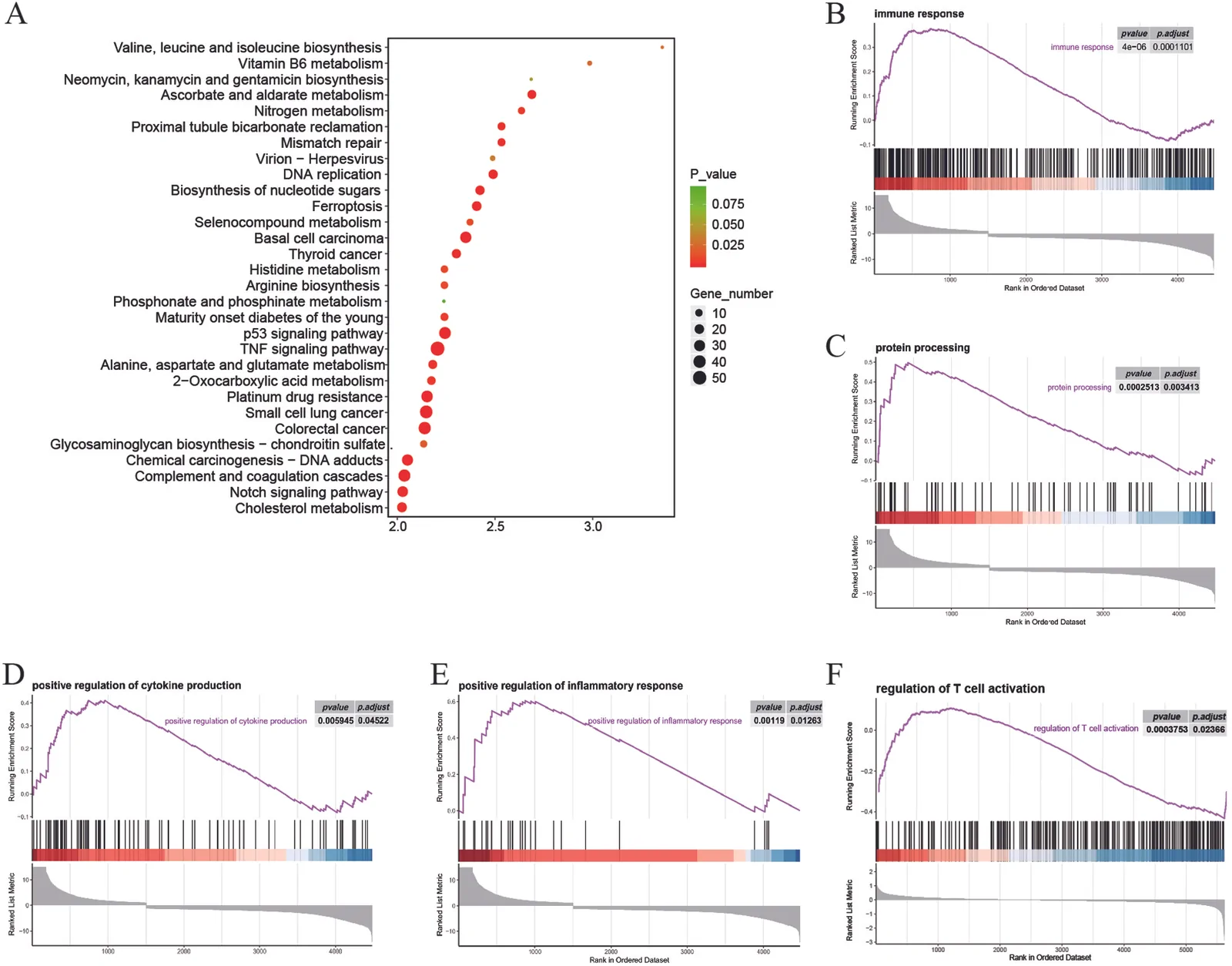
KEGG and GSEA enrichment analyses of DEGs following HHLA2 knockdown. **(A)** KEGG enrichment analysis showing pathways significantly influenced by HHLA2 knockdown. **(B–F)** GSEA enrichment analysis illustrating pathways that were upregulated following HHLA2 knockdown.

### 3.8 The potential signaling pathway of the HHLA-KCTD12 axis in regulating the immune microenvironment of LSCC

Spearman correlation analysis was performed to evaluate the associations between the expression of HHLA2 and KCTD12 and TNF family genes in 110 LSCC patients from the TCGA database. The results showed that the expression of HHLA2 and KCTD12 was significantly correlated with the expression of TNF, TNFSF10, and TNFSF14. In addition, it was also found to be significantly correlated with the expression of NF-κB1 (**Figure 8A**).

**Figure 8:**
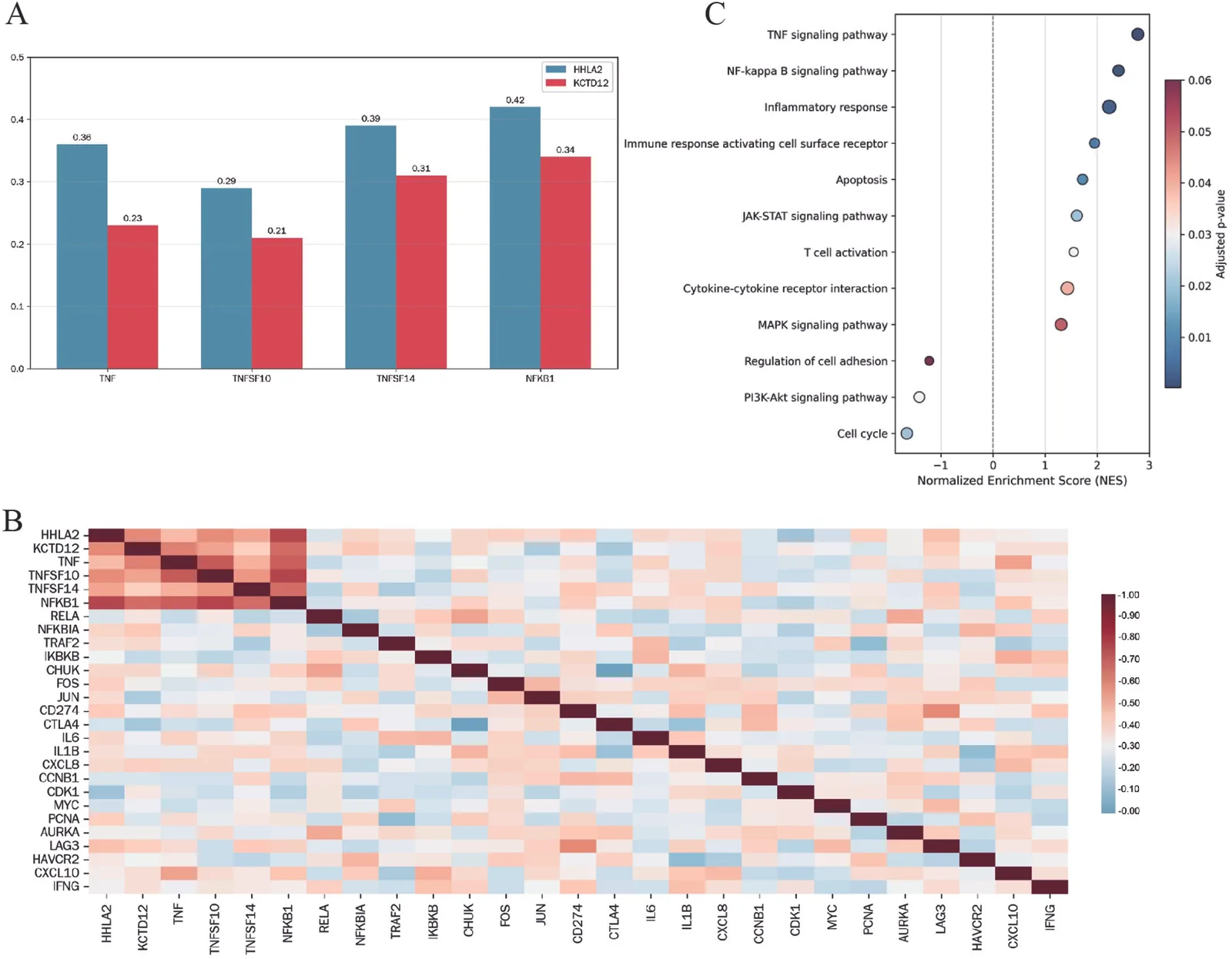
Analysis of the signaling pathway associated with the HHLA2-KCTD12 axis. **(A)** Spearman correlation analysis of HHLA2, KCTD12, and TNF family genes. **(B)** Heatmap clustering analysis reveals that the HHLA2/KCTD12 regulatory axis forms functionally synergistic co-expression modules with the TNF family, core genes of the NF-κB pathway, immune checkpoints, inflammatory factors, and proliferation-related genes, respectively.**(C)** GSEA enrichment analysis shows the enrichment status of different signaling pathways.

Heatmap clustering analysis of single-cell data of LSCC from the GSE234933 database. The results revealed that HHLA2 exhibited strong correlations with KCTD12, key TNF family ligands TNF, TNFSF10, TNFSF14 and NF-κB1(a core transcription factor of the NF-κB pathway). These findings suggest a coordinated expression pattern of these genes in LSCC tissues, implying that the HHLA2/KCTD12 regulatory axis may participate in the tumorigenesis and progression of LSCC via the TNF—NF-κB pathway. HHLA2 showed no significant strong correlations with traditional immune checkpoint molecules CTLA4, LAG3, HAVCR2, suggesting that it may exert immunosuppressive effects in LSCC through a regulatory axis independent of the PD-1/CTLA-4 pathway, thereby providing a potential novel target for immunotherapy in LSCC. Core genes of the TNF—NF-κB pathway exhibited moderate correlations with pro-inflammatory cytokines such as IL6, IL1B, and CXCL8, indicating that this pathway may contribute to immune evasion in LSCC by modulating the inflammatory microenvironment (**Figure 8B**).

GSEA enrichment analysis revealed significant enrichment of the TNF signaling pathway, NF-κB signaling pathway, inflammatory response, and immune activation cell surface receptor pathways (**Figure 8C**). This showed that the HHLA2/KCTD12 regulatory axis was highly associated with the TNF—NF-κB signaling pathway and may participate in the progression of LSCC by regulating inflammatory immune responses.

## 4 Discussion

HHLA2 expression in many tumors has been shown to facilitate tumor cell proliferation, migration, and invasion (17). In pancreatic cancer (PC), HHLA2 deletion can suppress PC cell migration and invasion by modulating the EMT process, the epidermal growth factor receptor (EGFR)/MAPK/ERK signalling pathway, and the mTOR/AKT pathway (18). In ovarian cancer (OC), HHLA2 may inhibit OC progression through activation of the NF-κB signalling pathway coupled with suppression of CA9 expression (19). HHLA2 is highly expressed in hepatocellular carcinoma (HCC) tumor tissues and correlates with poor tumor differentiation. Downregulation of SPP1 can counteract the proliferation, migration, and invasion of HCC cells induced by HHLA2 overexpression, thereby further validating the role of HHLA2 in promoting liver cancer cell growth and invasiveness through the SPP1/PI3K/AKT signalling axis (20). Additional studies in HCC have demonstrated that HHLA2 stabilises the hepatocyte growth factor receptor (c-Met) on the cell membrane, sustains downstream signal transduction, and consequently drives persistent tumor proliferation, invasion, and angiogenesis (21). *In vitro* studies on gallbladder cancer (GBC) have demonstrated that HHLA2 depletion suppresses TGF-β1- or lncRNA H19-induced GBC migration, invasion, proliferation, and EMT (22). HHLA2 contributes to tumorigenesis in clear cell renal cell carcinoma (ccRCC) via the METTL3/HHLA2 axis (23). Despite its recognized involvement in tumor progression, the molecular mechanisms by which HHLA2 regulates the biological behavior of LSCC remain unexplored.

In the present study, we provided the first evidence that HHLA2 expression was closely associated with KCTD12 and the TNF signaling pathway, as revealed through a combination of *in vitro* experiments and transcriptomic analyses. We systematically investigated the biological functions of HHLA2 in LSCC by performing a series of cellular functional assays, including the CCK-8 proliferation assay, scratch (wound healing) assay, and Transwell invasion assay. The results demonstrated that knockdown of HHLA2 in LSCC cell lines significantly impaired tumor cell proliferative, migratory, and invasive capabilities.

To elucidate the specific molecular mechanisms by which HHLA2 regulates the cytological functions of LSCC, we conducted transcriptomic sequencing and identified genes commonly downregulated in both TU177 and TU686 cell lines following HHLA2 knockdown. Besides that, analysis of human laryngeal carcinoma datasets identified KCTD12 as a critical target through which HHLA2 regulated LSCC biological functions. Building on this, KCTD12-overexpressing and HHLA2-knockdown LSCC cell lines were established via lentiviral transduction. Overexpression and knockdown efficiencies were validated by qPCR and Western blotting analysis, confirming the successful generation of stable cell lines. Functional assays, including CCK-8 proliferation, wound healing, and Transwell invasion assays, revealed that KCTD12 overexpression markedly rescued the inhibitory effects of HHLA2 knockdown on proliferation, migration, and invasion in TU177 and TU686 cells. These findings underscored the pivotal role of KCTD12 in mediating HHLA2-driven regulation of LSCC cell biological behavior.

KCTD12 is a member of the KCTD family, which comprises 26 distinct proteins. Functioning as part of a receptor complex, KCTD12 primarily interacts with the gamma-aminobutyric acid-B (GABA-B) receptor, thereby mediating K^+^ current desensitisation upon GABA-B receptor activation (16, 24). Accumulating evidence has increasingly suggested that KCTD12 may play a pivotal role in regulating the biological behaviors of tumor cells. Abbaszadegan MR et al. have reported that KCTD12 may exert tumor-suppressive effects in esophageal squamous cell carcinoma (ESCC) by inhibiting the Wnt/Notch signalling pathway, as well as stem cell-related factors and chromatin remodelling proteins (25). Li et al. have demonstrated that silencing KCTD12 enhances CRC cell stemness via activation of the extracellular signal-regulated kinase (ERK) pathway (26). Shen et al. have found that KCTD12 depletion promotes tumor growth and metastasis in both *in vitro* and *in vivo* melanoma models. Specifically, KCTD12 downregulation drives differentiated melanoma cells toward a more immature, highly tumorigenic state through the KCTD12-MITF-CD27 axis, and inhibition of KCTD12 further enhances melanoma cell stemness via interaction with CD271 (tumor necrosis factor receptor superfamily member 16) (27). KCTD12 has been reported to promote tumor initiation and proliferation by regulating the tumor cell cycle (28, 29). Moreover, its expression positively correlates with PD-1 and PD-L1 levels in breast cancer (30). Analysis of TCGA data has further revealed that KCTD12 expression is closely associated with the infiltration of both CD8^+^ and CD4^+^ T cells across a broad spectrum of tumor types (31, 32). Collectively, these findings indicate that KCTD12 may not only regulate intrinsic tumor cell functions but also contribute to shaping the tumor immune microenvironment.

To further dissect the molecular mechanisms underlying HHLA2-KCTD12 axis mediated regulation in LSCC, the study performed GO, KEGG, and GSEA enrichment analyses on the significantly up- and down-regulated differentially expressed genes (DEGs) between the experimental group and the control group. GO enrichment revealed that HHLA2 primarily participates in pathways related to amino acid transport and metabolism, the Wnt signaling pathway, and the EMT pathway, underscoring its multifaceted role in LSCC cell biology. GSEA further indicated that HHLA2 silencing resulted in pronounced downregulation of Wnt signaling–associated pathways involved in cell development, differentiation, angiogenesis, migration, and intercellular interactions. KEGG enrichment analysis demonstrated that HHLA2 predominantly participates in key signaling cascades, including the p53, TNF, and Notch pathways, with the TNF pathway exhibiting the most pronounced enrichment. Subsequently, HHLA2 suppression was associated with upregulation of inflammatory responses, cytokine production, antigen presentation, and other immune-related processes through further analysis using GSEA, highlighting its dual role in modulating both tumor-intrinsic and immune-related functions. Further analysis of the single-cell dataset of LSCC revealed that HHLA2 and KCTD12 are significantly correlated with the TNF family members TNF, TNFSF10, and TNFSF14, as well as with NFKB1. GSEA enrichment analysis also indicated that the signaling pathways were mainly enriched in the TNF signaling pathway and the NF-κB signaling pathway. This further confirms the molecular mechanism by which the HHLA2-KCTD12 axis regulates the tumor immune microenvironment through the TNF/NF-κB pathway in LSCC.

Recent studies have demonstrated that HHLA2 facilitates tumor progression by modulating the functions of immune cells within the TME (33). Elevated HHLA2 expression has been linked to immune cell infiltration, including MDSCs, CAFs, TAMs, and Tregs, as well as various chemokines such as CCL2, CCL20, and CXCL2 (34). Several studies have further demonstrated that HHLA2 expression is associated with the infiltration of CD8^+^ T cells and PD-1^+^ T cells (34–37). Beyond its effects on T cells, HHLA2 has also been linked to natural killer (NK) cell function; in chronic myeloid leukemia and lung cancer, HHLA2 has been shown to suppress NK cell cytotoxicity (38, 39). *In vitro* studies have shown that HHLA2 upregulation can induce M2 polarisation and chemotactic migration of macrophages via the IFN-γ/IFR1/HHLA2 axis, which in turn promotes PD-L1 expression in HCC cells, facilitating immune evasion and tumor progression (40). HHLA2/PD-L1 co-expression in chordoma has been found to independently predict patient prognosis (41).

The TNF signaling pathway plays a central role not only in shaping the tumor immune microenvironment and mediating antitumor immune responses (42, 43), but emerging evidence also suggests that the Notch–TNF axis regulates arterial valve EMT by balancing cell proliferation and apoptosis (44). Additionally, activation of the Wnt signaling pathway can elevate the pro-inflammatory cytokine TNF-α, potentially contributing to nucleus pulposus cell degeneration (45). Our findings suggested that HHLA2-KCTD12 axis might through TNF—NF-κB signaling pathway promoting malignant phenotypes in LSCC cells and remodeling the tumor immune microenvironment. this mechanistic insight not only advanced the understanding of LSCC biology but also provided a robust experimental foundation and theoretical rationale for the development of novel therapeutic strategies. However, this finding still requires further validation through additional studies at the mRNA and/or protein level, and future studies incorporating molecular and protein-level validation will still be necessary to establish the causal relationship between the HHLA2–KCTD12 axis and TNF–NF–κB signaling in LSCC.

## 5 Conclusion

In summary, our study identified HHLA2 as a pivotal regulator of LSCC progression through both tumor-intrinsic and immune-mediated mechanisms, established HHLA2 as a potential prognostic biomarker and therapeutic target in LSCC. It is proposed for the first time that the HHLA2-KCTD12 axis may promote tumor invasion and actively reshape the immune microenvironment via the TNF—NF-κB signaling pathway, while also influencing immune-related processes including cytokine production, antigen presentation, and inflammatory responses.

## Data Availability

The datasets presented in this study can be found in online repositories. The names of the repository/repositories and accession number(s) can be found in the article/**Supplementary Material**.
