# Supplementary material for: HHLA2-KCTD12 axis: a potential mechanism regulating the biological functions of laryngeal squamous cell carcinoma
Source: Front Oncol. 2026 Oct 8;16:1898263. doi: 10.3389/fonc.2026.1898263 (PMC13687375; doi:10.3389/fonc.2026.1898263)
Supplement: Supplementary file 1 [file Table1.docx]

Supplementary Table 1 The primer sequences used in the study

| **Gene** | **Forward (5’-3’)** | **Reverse (5’-3’)** |
| --- | --- | --- |
| HHLA2 | TACAAAGGCAGTGACCATTTGG | AGGTGTAAATTCCTTCGTCCAGA |
| KCTD12 | GCTCGGGCTACATCACCATC | GGTCCCGGCTTTCGTTCAG |
| GAPDH | GGAGCGAGATCCCTCCAAAAT | GGCTGTTGTCATACTTCTCATGG |
